# Felty Syndrome–Associated Clonal Cytotoxic T‐Cell Disease With Terminal Aggressive Progression: Shared *STAT3* and *TET2* Mutations Across a Prolonged Disease Course

**DOI:** 10.1002/jha2.70406

**Published:** 2026-09-28

**Authors:** Kei Saito, Akihiko Yokohama, Oh Kwan Ee, Momoka Sorimachi, Kenji Imamura, Nobuhiko Kobayashi, Yuri Miyazawa, Yoshiyuki Ogawa, Takayuki Saito, Takahiro Shirakura, Sumihito Nobusawa, Hiroshi Handa

**Affiliations:** ^1^ Department of Hematology Gunma University Graduate School Medicine Meabashi Japan; ^2^ Department of Hematology Gunma Prefectural Cancer Center Ohta Japan; ^3^ Division of Transfusion Service Gunma University Hospital Faculty of Medicine Gunma University Meabashi Japan; ^4^ Department of Laboratory Sciences Gunma University Graduate School of Health Sciences Meabashi Japan; ^5^ Clinical Department of Pathology Gunma University Hospital Maebashi Japan; ^6^ Department of Human Pathology Gunma University Graduate School of Medicine Meabashi Japan

**Keywords:** Felty syndrome, *STAT3*, T‐cell large granular lymphocytic leukaemia, *TET2*, γδ T‐cells

## Abstract

**Introduction:**

Biological overlap between Felty syndrome (FS) and clonal cytotoxic T‐cell disorders is increasingly recognized, but aggressive progression is uncommon.

**Methods:**

We investigated an autopsy‐confirmed case of terminal aggressive clonal T‐cell disease that developed after a 4‐year clinical course diagnosed as FS using serial immunophenotypic and molecular analyses.

**Results:**

Evolving γδ T‐cell populations, TCR clonality, shared *STAT3* and *TET2* mutations, and a newly emergent complex karyotype supported clonal continuity and terminal aggressive progression.

**Conclusion:**

This case provides clinicopathological evidence of long‐term evolution in FS‐associated clonal cytotoxic T‐cell disease.

**Trial Registration:**

The authors have confirmed clinical trial registration is not needed for this submission

1

**Felty** syndrome (FS) is an uncommon extra‐articular manifestation of rheumatoid arthritis (RA), classically defined by the triad of RA, neutropenia, and splenomegaly. Growing evidence suggests biological overlap between FS and clonal cytotoxic T‐cell disorders, particularly T‐cell large granular lymphocytic leukaemia (T‐LGLL), with recurrent activating *STAT3* mutations identified in both conditions [1, 2, 3, 4]. However, the long‐term evolution of FS‐associated disease into an aggressive clonal T‐cell process remains poorly characterized. Here, we report an autopsy‐confirmed case with late emergence of T‐cell clonality and shared *STAT3* and *TET2* mutations suggesting clonal continuity before a terminal aggressive phase.

A 74‐year‐old woman was diagnosed with RA 14 years before death; RF was negative at 2 IU/mL, while anti‐CCP antibody testing was not documented. Mild splenomegaly was first documented 8 years before death and progressed thereafter. She had initially presented 6 years before death with direct antiglobulin test‐positive autoimmune haemolytic anaemia, splenomegaly and neutropenia (Figure 1A). Given the clinical concern regarding lymphoma in the setting of splenomegaly and cytopenias, splenic needle biopsy and bone marrow examination were performed. However, neither demonstrated overt lymphoma; T‐cell receptor (TCR) gene rearrangement analysis was not performed on this initial bone marrow specimen. A retrospective review of the initial data from splenic flow cytometry demonstrated a γδ T‐cell population accounting for approximately 11% of analysed cells (Figure S1A). Prednisolone therapy led to improvements in both anaemia and neutropenia. Neutropenia recurred during steroid tapering, but repeat bone marrow examination again showed no clear abnormalities, with no evidence of clonal T‐cell proliferation. On the basis of the identified RA, neutropenia, and splenomegaly, she was subsequently diagnosed with FS 4 years before death.

**FIGURE 1:**
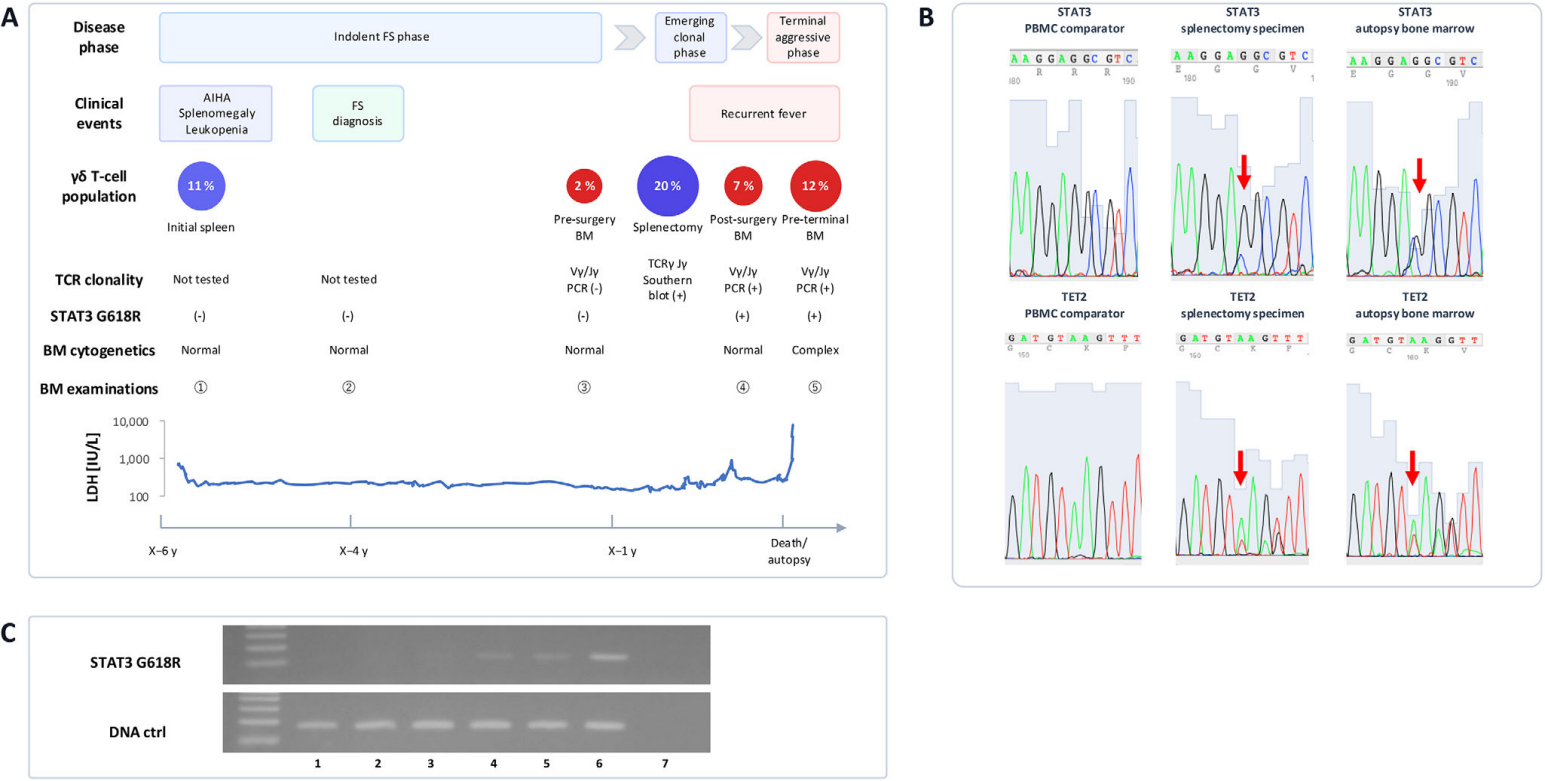
Clinical course and shared *STAT3* and *TET2* mutations in Felty syndrome‐associated clonal cytotoxic T‐cell disease with terminal aggressive progression. (A) Clinical timeline illustrating disease phase, major clinical events, γδ T‐cell populations, TCR clonality, serial STAT3 p.G618R results, bone marrow cytogenetics and serum LDH over time. (B) Sanger sequencing chromatograms of *STAT3* and *TET2* in temporally separated specimens. Identical mutations are seen in the splenectomy and autopsy bone marrow specimens, but not in peripheral blood mononuclear cells used as a comparator. Red arrows indicate mutation sites. (C) Allele‐specific PCR for STAT3 p.G618R in serial archival bone marrow specimens. Lanes 1–5 correspond to bone marrow examinations 1–5 in panel A. Lane 6 represents the STAT3 p.G618R‐positive splenectomy specimen as a positive control, and lane 7 represents the negative water control. The lower panel shows amplification of the DNA control.

The disease course thereafter was relatively indolent for several years. Approximately 1 year before death, she began to experience recurrent febrile episodes, around the time of splenectomy and thereafter. Histological examination of the spleen showed congested red pulp containing small CD3‐positive lymphocytes without significant atypia. There was no clear predominance of CD4‐ or CD8‐positive lymphocytes, and many lymphocytes expressed TIA‐1. No overt lymphoma was identified (Figure S2A,B). In contrast, flow‐cytometric analysis of splenic tissue at splenectomy identified an expanded γδ T‐cell population, accounting for approximately 20% of analysed cells (Figure S1B). In addition, Southern blot analysis of the splenectomy specimen demonstrated clonal rearrangement of the TCR γ‐chain Jγ region, whereas no rearrangement of the TCR β‐chain Cβ1 region was detected. TCR gene rearrangement analysis had not been performed on the bone marrow specimens obtained at the initial presentation or during corticosteroid tapering. Polymerase chain reaction (PCR) analysis of a pre‐splenectomy bone marrow specimen showed no Vγ/Jγ rearrangement, whereas a subsequent bone marrow specimen obtained after splenectomy but before aggressive transformation was positive for Vγ/Jγ rearrangement and showed a normal karyotype on G‐banding (Figure 1A). Corresponding flow‐cytometric findings from the pre‐ and post‐splenectomy bone marrow specimens are shown in Figure S1C,D, respectively.

At the final admission, the patient displayed rapid systemic deterioration, including worsening constitutional symptoms and haematological abnormalities. Serum concentrations of lactate dehydrogenase (LDH) rose rapidly during the terminal phase, ultimately exceeding 8000 U/L (reference range, 124–229 U/L). Peripheral blood examination showed an increase in markedly atypical lymphoid cells, some of which displayed conspicuous cytoplasmic granules (Figure S2C). Bone marrow examination performed several days before death demonstrated a γδ T‐cell population accounting for approximately 12% of analysed cells; Vγ/Jγ rearrangement remained detectable (Figure 1A, Figure S1E). Cytogenetic analysis at this time revealed a complex karyotype that had not been detected in the preceding post‐splenectomy marrow: 49, X, idic(X)(q11), dup(1), add(2)(q11.2), add(5)(q11.2), +9, +mar1×2[4]/46, XX[16]. The patient died despite supportive management.

Autopsy performed to clarify the nature of the terminal disease demonstrated widespread infiltration by atypical T cells. Involvement of the liver and uterus was particularly prominent, along with involvement of the bone marrow and other sites (Figure S3A). Histologically, the infiltrate comprised medium‐sized atypical lymphoid cells with irregular and pleomorphic nuclei (Figure S3B). Immunophenotypically, tumour cells were CD3+, CD7+, TIA‐1+, CD5−, CD4− and CD8− (Figure S3C). Taken together, these findings supported terminal aggressive progression of a clonal cytotoxic T‐cell neoplasm, although definitive subclassification remained uncertain [5].

To investigate the molecular basis of disease progression, whole‐exome sequencing (WES) was performed using DNA extracted from autopsy‐derived bone marrow tissue (Agilent SureSelect Human All Exon V6 and NovaSeq 6000; Illumina, San Diego, CA, USA). WES identified somatic mutations in *STAT3* (c.1852G>C, p.G618R; variant allele frequency [VAF], 38.2%) and *TET2* (c.3893_3894insT, p.C1298fs; VAF, 31.9%). The *STAT3* variant was located within the SH2 domain, a functionally relevant region in which recurrent activating mutations have been described in T‐LGLL [3, 6]. The *TET2* alteration was a frameshift mutation predicted to result in loss of function. No pathogenic *TP53* alteration was identified.

To determine whether these mutations were restricted to the terminal aggressive phase, Sanger sequencing was performed on DNA extracted from her non‐leukaemic peripheral blood mononuclear cells (PBMCs, used as a peripheral blood comparator sample) because non‐haematopoietic tissue was unavailable. Complete exclusion of low‐level blood‐derived mutant clones, including age‐related clonal haematopoiesis, thus could not be assured. Identical *STAT3* and *TET2* mutations were detected in both the splenectomy and autopsy bone marrow specimens, whereas these alterations were not detected in the PBMC comparator (Figure 1B). Retrospective allele‐specific PCR of serial archival bone marrow specimens showed that STAT3 p.G618R was undetectable in examinations 1–3 but detectable in examinations 4 and 5, coinciding with the emergence of Vγ/Jγ rearrangement (Figure 1A,C).

This case documents a prolonged clinical course initially diagnosed as FS followed by terminal aggressive clonal T‐cell disease. Although FS and clonal cytotoxic T‐cell disorders are increasingly recognized to overlap biologically, overt aggressive progression appears uncommon [7, 8, 9, 10]. Similar diagnostic difficulty has been described in non‐leukaemic or splenic‐predominant T‐LGLL variants associated with RA, neutropenia, and splenomegaly, in which clonal T‐cell disease may clinically mimic FS [2].

Clonal evolution became more apparent only during the later disease phase. Early bone marrow examinations did not demonstrate any overt lymphoma or clonal T‐cell proliferation. However, retrospective review showed that a γδ T‐cell population was already detectable in the initial splenic specimen. In the spleen, this population accounted for approximately 11% and 20% of all analysed nucleated cells in the initial biopsy and splenectomy specimen, respectively; a γδ T‐cell population was also detected in bone marrow several days before death. These values are not directly comparable with published reference ranges based on gated lymphocytes or T cells and do not precisely quantify clonal burden [11, 12]. In parallel, TCRγ clonality became demonstrable by Southern blot and later by PCR, further supporting stepwise emergence of an overt clonal T‐cell process [1, 2, 3, 4, 7, 8, 9, 10].

The molecular findings are also notable. Prior studies have highlighted overlap between FS and T‐LGLL, including recurrent *STAT3* mutations in a subset of cases [1, 2, 3, 4, 6]. The present case is unusual from the perspective of the very long clinical course followed by terminal aggressive progression. The VAFs of 38.2% and 31.9% indicate that both mutations were present in a substantial proportion of the terminal neoplastic clone. Retrospective analysis of serial bone marrow specimens showed that *STAT3* p.G618R became detectable only in the later disease phase, temporally coinciding with the emergence of TCR clonality. A newly emergent complex karyotype was also identified shortly before death, consistent with additional genomic evolution during terminal progression [8, 9].

The coexistence of *STAT3* and *TET2* mutations is insufficient to establish a direct mechanism underlying terminal aggressive progression, and such mutations may occur in chronic clonal T‐cell disorders without aggressive behaviour [4]. No *TP53* alteration was identified, although *TP53* abnormalities have been implicated in aggressive progression in some T‐LGLL‐related disorders [8, 9]. Nevertheless, detection of the same mutations in temporally separated specimens supports clonal continuity rather than an unrelated terminal process [7, 8, 9, 10]. This case is therefore most informative as a clinicopathological illustration of long‐term evolution of FS‐associated disease with terminal aggressive clonal T‐cell proliferation.

The reporting of this case has some limitations. Archival specimens from the earlier disease phase were limited, precluding comprehensive high‐depth molecular analysis and quantitative comparison of clonal burden over time. TCRδ gene rearrangement analysis was not performed; therefore, clonal continuity could not be assessed at the TCRδ locus. Although the pathological diagnosis was aggressive T‐cell lymphoma, definitive phenotypic subclassification of the autopsy lesion was not possible [5]. Nonetheless, serial clinical observations, evolving TCR clonality, shared mutations across temporally separated specimens, and terminal pathological confirmation support stepwise disease progression.

In summary, we have reported a case of autopsy‐confirmed terminal aggressive clonal T‐cell disease arising after a prolonged clinical course initially diagnosed as FS. Shared *STAT3* and *TET2* mutations in pre‐terminal and terminal specimens suggest that a biologically relevant clonal T‐cell population may precede overt aggressive progression by years. This case highlights the need for careful re‐evaluation of FS‐associated disease when new systemic symptoms, rising LDH, or emerging haematologic abnormalities develop, although further studies are required to clarify the biological relationship and long‐term behaviour of FS‐associated clonal cytotoxic T‐cell disorders.

## Author Contributions

K.S. and A.Y. collected and interpreted the clinical data, performed experiments, reviewed the literature and drafted the manuscript. O.K., M.S., K.I., N.K., Y.M. and Y.O. contributed to patient management and data collection. T. Shirakura and S.N. reviewed and interpreted the pathological findings. T. Saito contributed to data interpretation and critical revision of the manuscript. H.H. supervised the study and critically revised the manuscript. All authors approved the final version of the manuscript.

## Funding

Akihiko Yokohama received research funds from Kyowa Kirin Co. Ltd. and Chugai Pharmaceutical Co. Ltd.

## Ethics Statement

An opt‐out informed consent protocol was used for this study. This study and the consent procedure were reviewed and approved by the institutional review board of Gunma University (approval no. HS2020‐103). The study was conducted in accordance with the Declaration of Helsinki.

## Conflicts of Interest

The authors declare no conflicts of interest.

## Supporting information




**Supporting File 1**: jha270406‐sup‐0001‐FigureS1‐S3.pptx

## Data Availability

The data that support the findings of this study are openly available in the Japanese Genotype‐phenotype Archive (JGA) (Accession #: JGAS000916).
